# Differential expression and predictive value of monocyte scavenger receptor CD163 in populations with different tuberculosis infection statuses

**DOI:** 10.1186/s12879-019-4525-y

**Published:** 2019-11-28

**Authors:** Qianqian Liu, Qinfang Ou, Huaxin Chen, Yan Gao, Yuanyuan Liu, Yuzhen Xu, Qiaoling Ruan, Wenhong Zhang, Lingyun Shao

**Affiliations:** 1 Department of Infectious Diseases, Huashan Hospital, Fudan University, 12 Wulumuqi Zhong Road, Shanghai, 200040 China; 2 Department of Pulmonary Diseases, Wuxi Infectious Diseases Hospital, Wuxi, 214005 China; 3 Key Laboratory of Medical Molecular Virology, Ministry of Education and Health, Shanghai Medical College, and Institutes of Biomedical Science, Fudan University, Shanghai, 200032 China; 4 State Key Laboratory of Genetic Engineering, School of Life Science, Fudan University, Shanghai, 200438 China; 5 National Clinical Research Center for Aging and Medicine, Huashan Hospital, Fudan University, Shanghai, 200040 China

**Keywords:** Tuberculosis, Monocyte subpopulations, CD163, Innate immunity, Disease severity

## Abstract

**Background:**

Monocytes are the predominant innate immune cells at the early stage of *Mycobacterium tuberculosis* (*M. tb*) infection as the host defense against intracellular pathogens. Understanding the profile of different monocyte subpopulations and the dynamics of monocyte-related biomarkers may be useful for the diagnosis and prognosis of tuberculosis.

**Methods:**

We enrolled 129 individuals comprising patients with pulmonary tuberculosis (PTB) (*n* = 39), tuberculous pleurisy (TBP) (*n* = 28), malignant pleural effusion (MPE) (*n* = 21), latent tuberculosis infection (LTBI) (*n* = 20), and healthy controls (HC) (*n* = 21). Surface expression of CD14, CD16, and CD163 on monocytes was detected using flow cytometry. In addition, soluble CD163 (sCD163) was determined by enzyme linked immunosorbent assay.

**Results:**

Higher frequency of CD14^+^CD16^+^ (15.7% vs 7.8%, *P* < 0.0001) and CD14^−^CD16^+^ (5.3% vs 2.5%, *P* = 0.0011) monocytes and a decreased percentage of CD14^+^CD16^−^ (51.0% vs 70.4%, *P* = 0.0110) cells was observed in PTB patients than in HCs. Moreover, PTB patients displayed a higher frequency of CD163^+^ cells in CD16^+^ monocytes than those in the HC group (40.4% vs 11.3%, *P* < 0.0001). The level of sCD163 was elevated in TBP patients and was higher in pleural effusion than in plasma (2116.0 ng/ml vs 1236.0 ng/ml, *P* < 0.0001). sCD163 levels in pleural effusion and plasma could be used to distinguish TBP from MPE patients (cut-off values: 1950.0 and 934.7 ng/ml, respectively; AUCs: 0.8418 and 0.8136, respectively). Importantly, plasma sCD163 levels in TBP patients decreased significantly after anti-TB treatment.

**Conclusions:**

Higher expression of membrane and soluble CD163 in active tuberculosis patients might provide insights regarding the pathogenesis of tuberculosis, and sCD163 may be a novel biomarker to distinguish TBP from MPE and to predict disease severity.

## Background

*Mycobacterium tuberculosis* (*M. tb*) invades the host and activates immune responses including innate and adaptive immunity, and causes tuberculosis (TB), which remains a global public health concerns [1]. Monocytes/macrophages, as the first line of defense, are critical for host immunity against *M. tb* infection [2–4]. Human monocytes are classified into three major subpopulations based on the expression of markers CD14 and CD16: CD14^+^CD16^−^ (classical), CD14^+^CD16^+^ (intermediate), and CD14^−^CD16^+^ (non-classical) monocytes [5–7]. The three monocyte subsets represent different stages of macrophage differentiation and play different roles in *M. tb* infection [8]. Classical monocytes account for the majority of total subsets; they differentiate into pro-inflammatory M1 macrophages (classical activated) that are permissive to *M. tb* infection in vitro and produce several pro-inflammatory cytokines [8]. However, both non-classical and intermediate monocytes are considered precursors of anti-inflammatory M2 macrophages (alternative activated) in different disease conditions [8].

Increasing evidence suggests that switching the M1/M2 phenotype influences the clinical outcome of host infection with *M. tb* [2, 3, 9, 10]. The early stage of anti-TB immune responses is predominated by M1 macrophages, which are characterized by high production of iNOS and IFN-γ, with the function of killing most *M. tb* and restricting the replication of the remainder. Nevertheless, M2 macrophages are poorly microbicidal and play an immunomodulatory role [3]. Thus, the shift from M1 to M2 polarization during *M. tb* infection might be a microbial strategy to escape immune attack and cause disease progression.

CD163, a scavenger receptor that serves as an M2 macrophage phenotype marker, is also expressed on monocytes, and binds to haptoglobin-hemoglobin complexes, mediating their endocytosis [11]. In the context of TB, expansion of CD16^+^CD163^+^MerTK^+^ monocytes contribute negatively to the host defense against *M. tb* by a low ratio of pro−/anti-inflammatory cytokine production and a poor capacity to activate T cells. Moreover, CD163 and MerTK act as M2-like macrophage activation markers, which are characterized by pathogen permissivity and immunomodulatory activity [12]. Indeed, the soluble form of CD163 (sCD163) from monocyte activation, exists in plasma and is correlated with TB disease severity. In this study, we explored the expression of scavenger receptor CD163 on monocyte subsets in populations with different tuberculosis infection statuses including active tuberculosis, latent tuberculosis infection and non-infection, detected the sCD163 levels in plasma and pleural effusions, further assessed the value of sCD163 in diagnosing tuberculosis and in predicting the disease severity and treatment outcome.

## Methods

### Study population

In total, 129 individuals were enrolled in this study including patients with pulmonary TB (PTB) (*n* = 39), tuberculous pleurisy (TBP) (*n* = 28), malignant pleural effusion (MPE) (*n* = 21), latent tuberculosis infection (LTBI) (*n* = 20), and healthy controls (HC) (*n* = 21). All the patients were recruited from Wuxi Fifth People’s Hospital, Zhuji People’s Hospital, and Fudan University Affiliated Huashan Hospital from 2011 to 2018. Populations with LTBI and HC were recruited from the relatives of PTB patients and the volunteers of Huashan Hospital during the same period.

This study was approved by the Ethics committee of Huashan Hospital, Fudan University. Written informed consent was obtained from all the participants.

### Diagnosis criteria

PTB patients were diagnosed based on identification of *M. tb* in the sputum or bronchoscopy. TBP patients were diagnosed according to the following criteria: 1) pleural biopsy; 2) acid-fast bacilli (AFB) smear or culture positive in pleural effusion (PE); 3) AFB smear or culture positive in sputum; 4) combination with clinical symptoms, radiological results, and effective anti-TB treatment upon lack of etiology evidence. MPE patients without tuberculosis infection were enrolled as controls, diagnosed based on either histopathology in pleural tissue or cytology in PE. Populations with LTBI and HC were interferon-γ release assay (IGRA)-positive and -negative, respectively. In addition, they had no evidence of active tuberculosis infection.

All enrolled participants were free of HIV infection, autoimmune disease or other chronic infections (i.e., chronic HBV/HCV infection). Furthermore, they were not undergoing immune-modulating treatment.

### Interferon-γ release assay (IGRA)

In this study, the T-cell-based enzyme-linked immunospot assay for tuberculosis (T-SPOT.TB) (Oxford Immunote Ltd., Oxford, UK) was performed as IGRA according to the manufacturer’s instructions. The positive results were analyzed as described previously [13].

### Cell surface staining and flow cytometry

Peripheral blood mononuclear cells (PBMCs) were separated by Ficoll density-gradient centrifugation from 10 ml venous blood samples. For phenotyping of monocyte subsets, one million fresh PBMCs were surface stained with monoclonal anti-human CD14-APC (61D3, eBioscience), CD16-FITC (3G8, Biolegend) and CD163-BV421 (GHI/61, Biolegend) at room temperature in the dark for 15 min, and were washed twice in PBS containing 2% FBS. Stained samples were detected on a Beckman Moflo flow cytometer. According to the expression of CD14 and CD16, three major monocyte subsets in PTB patients and controls were analyzed as classical (CD14^+^CD16^−^), intermediate (CD14^+^CD16^+^), and non-classical (CD14^−^CD16^+^) monocytes. Furthermore, expression of the scavenger receptor CD163 on monocytes subsets was evaluated. Data were analyzed using Flowjo 10 (Tree Star, Inc. Ashland, OR).

### Detection of soluble CD163 levels using enzyme linked immunosorbent assay (ELISA)

Levels of soluble (s)CD163 were assessed in plasma and pleural effusion (PE) samples stored at − 80 °C using an ELISA kit (DC1630, R&D systems) according to the manufacture’s protocol. Absorbance was immediately determined at 450 nm and 570 nm on a microplate reader, and was corrected by subtracting the readings at 570 nm from the readings at 450 nm. The minimum detectable dose was 0.177 ng/ml.

### Statistical analysis

Statistical analysis was performed using GraphPad Prism 6 (GraphPad Software, Inc. La Jolla, CA) and MultiExperiment Viewer (MeV) 4.9 (Dana-Farber Cancer Institute, Boston, USA). Two different groups were compared using an unpaired *t* test or Mann-Whitney *U* test (when the variances were significantly different). Categorical variables were compared using the *χ*^*2*^ test or Fisher’s exact test, as appropriate. Receiver operating characteristics (ROC) curve analysis was performed to evaluate the diagnostic performance of sCD163 levels in plasma or the PE for differential diagnosis of PTB and controls, or TBP and MPE. Comparisons of plasma sCD163 levels in unpaired pre- and post-treatment groups were performed using unpaired Mann-Whitney *U* test. Statistical significance was referred as *P* < 0.05.

## Results

### Clinical characteristics of participants

The 129 enrolled individuals were divided into five groups, as shown in Table 1. Of the PTB group (*n* = 39), 35 confirmed cases based on sputum AFB smear positive or culture positive for *M. tb*, and 4 patients diagnosed by bronchoscopy were included. Among 28 patients with TBP, more than one quarter (28.6%) had pulmonary TB simultaneously, and 11 (39.3%) were confirmed by pleural biopsy (*n* = 8) or by identification of AFB in pleural effusions (*n* = 3). In this study, nearly half of the active tuberculosis (ATB) patients (*n* = 31, 46.3%) were anti-TB treatment naïve at recruitment. The characteristics of all individuals are presented in Table 1.

**Table 1:** Clinical characteristics of enrolled individuals

	ATB (*n* = 67)		MPE (*n* = 21)	LTBI (*n* = 20)	HC (*n* = 21)	*P* value
	PTB (*n* = 39)	TBP (*n* = 28)				
Male/Female	30/9	23/5	16/5	10/10	13/8	0.0992
Age, median (IQR)	44 (28–62)	48 (23–66)	72 (63–78)	53 (40–64)	46 (27–65)	<0.0001
History of BCG vaccination, n (%)	24 (61.5)	19 (67.8)	11 (52.4)	15 (75.0)	17 (80.9)	0.2913
IGRA+, n (%)	/	/	/	20 (100.0)	0 (0)	/
Sputum AFB smear or culture positive, n (%)	35 (89.7)	8 (28.6)	0	/	/	/
1+	11 (28.2)	7 (25.0)	0	/	/	/
>1+ (2+, 3+, 4+)	24 (61.5)	1 (3.6)	0	/	/	/
Cavity, n (%)	19 (48.7)	1 (3.6)	0	/	/	/
*M. tb* detection in pleural effusion						
Confirmed TBP by pleural biopsy, n (%)	/	8 (28.6)	0	/	/	/
AFB smear or culture positive, n (%)	/	3 (10.7)	0	/	/	/
anti-TB therapy status at enrollment						
Naive, n (%)	8 (20.5)	23 (82.1)	/	/	/	/
Days on anti-TB, median (IQR)	2 (1–7)	9 (5–12)	/	/	/	/

Variables are shown as medians and IQRs. χ^2^ test was used for categorical data, and Kruskal-Wallis test was used for continuous data

*ATB* active tuberculosis, *PTB* pulmonary tuberculosis, *TBP* tuberculous pleurisy, *MPE* malignant pleural effusion, *LTBI* latent tuberculosis infection, *HC* healthy control, *IQR* interquartile range, *BCG* bacillus Calmette-Guerin, *IGRA* interferon-γ release assay, *AFB* acid-fast bacilli

### Increased percentage of circulating CD16^+^ monocytes and lack of classical (CD14^+^CD16^−^) monocytes in active tuberculosis patients

**Fig. 1:**
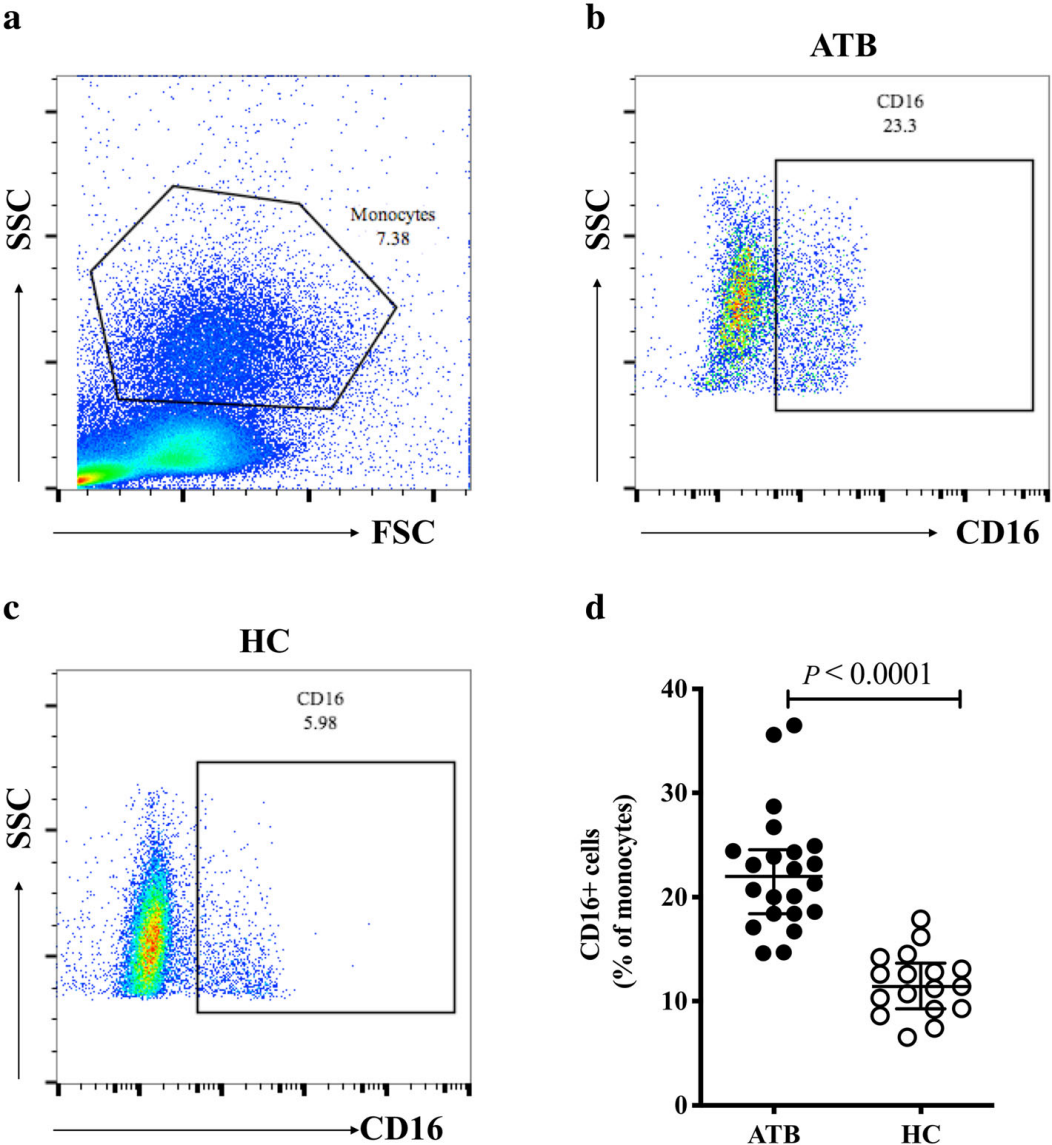
Expression of CD16 on monocytes in ATB and HC groups. **a** Monocytes were gated based on FSC and SSC dot plots. **b** Representative monocyte-gated dot plots of CD16 expression in ATB patients. **c** Representative monocyte-gated dot plots of CD16 expression in HC. **d** Percentages of CD16^+^ monocytes were compared between the ATB and HC groups. Data are expressed as median with IQR and are analyzed using Mann-Whitney test. ATB, active tuberculosis; HC, healthy control; FSC, forward scatter; SSC, side scatter; IQR, interquartile range

**Fig. 2:**
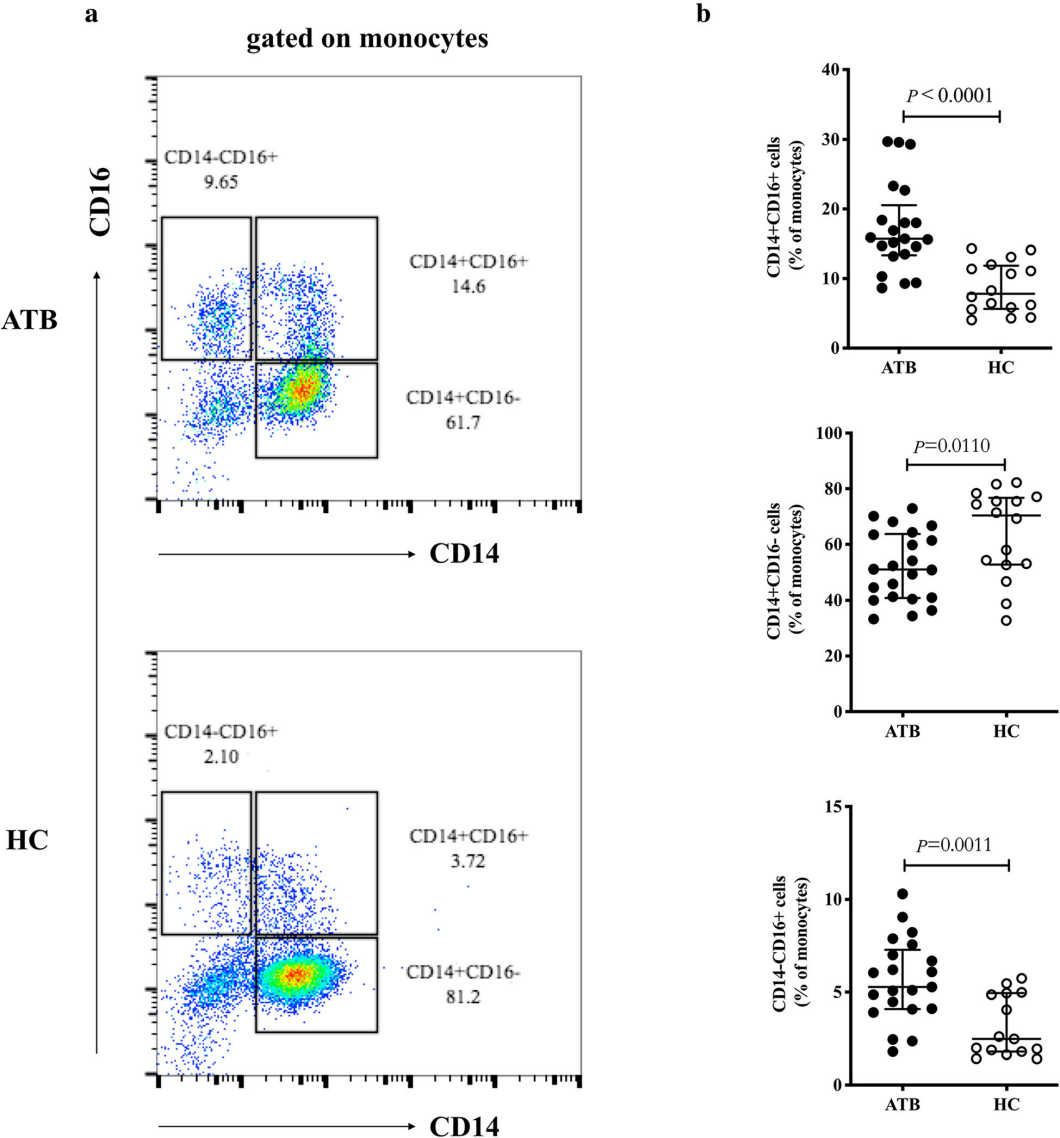
Monocyte subset profiles in ATB and HC groups. **a** Representative monocyte-gated dot plots showing the profile of monocyte subsets based on CD14 and CD16 expression in ATB patients and the HC group. **b** Percentages of CD14^+^CD16^+^ (top), CD14^+^CD16^−^ (center), and CD14^−^CD16^+^ (below) monocytes were compared between the ATB and HC group. Data are expressed as median with IQR and are analyzed using Mann-Whitney test. ATB, active tuberculosis; HC, healthy control; IQR, interquartile range

Given the importance of monocytes in the innate immune response to tuberculosis, we investigated the profiles of monocyte subsets in ATB patients based on the expression of CD14 and CD16. An evidently high expression of CD16 on monocytes was observed in the ATB group (Fig. 1b) compared to HC (Fig. 1c) (22.0% vs 11.4%, *P* < 0.0001, Fig. 1d). Furthermore, ATB patients exhibited higher percentages of CD14^+^CD16^+^ (15.7% vs 7.8%, *P* < 0.0001) and CD14^−^CD16^+^ (5.3% vs 2.5%, *P* = 0.0011) monocyte subsets than the HC group (Fig. 2a and b). Notably, in this study, the CD14^+^CD16^−^monocytes subset, considered as the precursor of M1 macrophages, was significantly reduced in patients with ATB compared with the HC group (51.0% vs 70.4%, *P* = 0.0110) (Fig. 2a and b).

### Higher expression of scavenger receptor CD163 on CD16^+^ monocytes in active tuberculosis patients

**Fig. 3:**
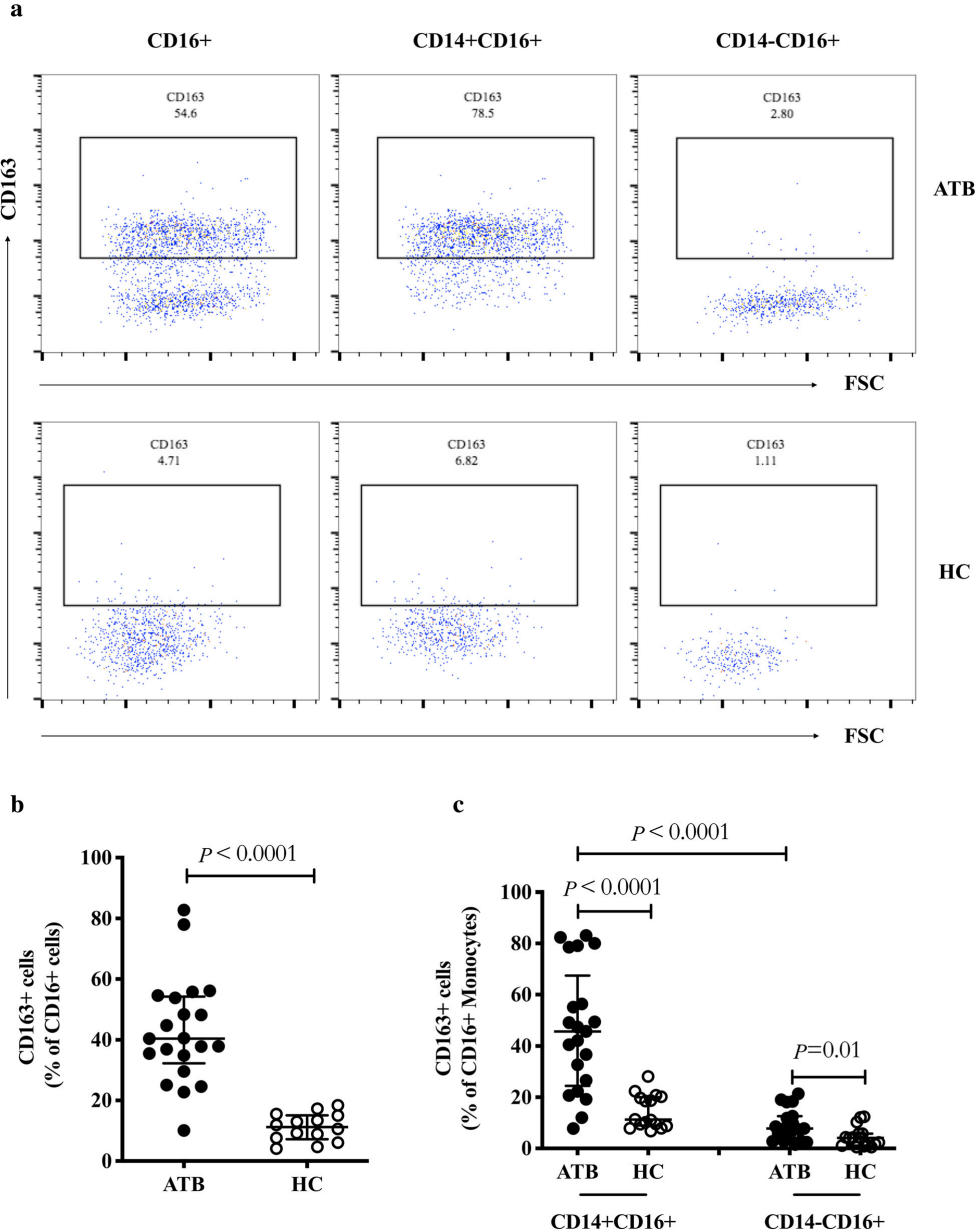
Expression of CD163 on CD16^+^ monocytes in ATB and HC groups. **a** Representative dot plots of CD163 expression on CD16^+^ (left), CD14^+^CD16^+^ (center), and CD14^−^CD16^+^ (right) monocytes in the ATB and HC groups. **b-c** Percentages of CD163^+^ cells among CD16^+^ (**b**), CD14^+^CD16^+^, and CD14^−^CD16^+^ monocytes (**c**) were compared between the ATB and HC groups. Data are expressed as median with IQR and are analyzed using Mann-Whitney test. ATB, active tuberculosis; HC, healthy control; IQR, interquartile range

Previously, the scavenger receptor CD163 was reported as a major biomarker of M2 macrophages [3], which play an anti-inflammatory role in tuberculosis infection. Thus, we hypothesized that CD163 was upregulated on the CD14^−^CD16^+^ subset, namely the precursor of M2 macrophages. To test this, we analyzed the frequency of CD163-expressing CD16^+^ monocytes including CD14^+^CD16^+^ and CD14^−^CD16^+^ subsets. As expected, ATB patients displayed a higher frequency of CD163^+^cells in CD16^+^ monocytes compared with the HC group (40.4% vs 11.3%, *P* < 0.0001, Fig. 3a and b), in both CD14^+^ (45.7% vs 11.3%, *P* < 0.0001, Fig. 3a and c) and CD14^−^ (7.8% vs 4.1%, *P* = 0.0100, Fig. 3a and c) subpopulations. Moreover, in ATB patients, we found that CD163 was mainly expressed on the CD14^+^CD16^+^ rather than the CD14^−^CD16^+^ subset (45.7% vs 7.8%, *P* < 0.0001, Fig. 3c).

### Soluble CD163 levels in plasma and pleural effusion and the value of sCD163 in diagnosing tuberculosis

**Fig. 4:**
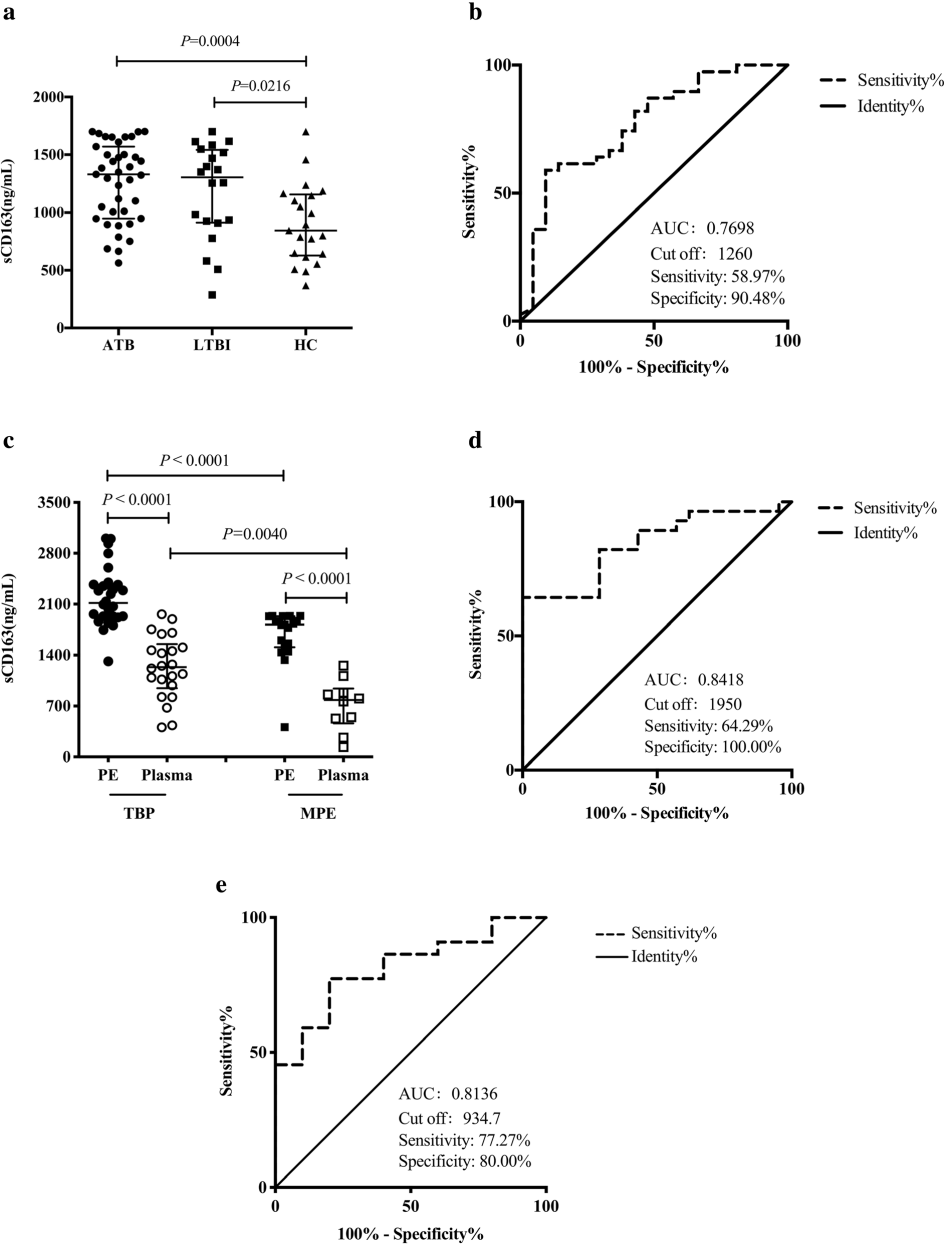
Levels of sCD163 in plasma and pleural effusion of individuals. **a** Expression of sCD163 in plasma of populations with different tuberculosis infection statuses. **b** ROC analysis of sCD163 in plasma for the differential diagnosis of PTB and HC. **c** Expression of sCD163 in the plasma and PE of TBP and MPE patients. **d-e** ROC analysis of sCD163 in PE (**d**) and in the plasma (**e**) for the differential diagnosis of TBP and MPE. Data are expressed as median with IQR and are analyzed using Mann-Whitney test. PTB, pulmonary tuberculosis; LTBI, latent tuberculosis infection; HC, healthy control; AUC, area under ROC curve; TBP, tuberculous pleurisy; MPE, malignant pleural effusion; PE, pleural effusion; IQR, interquartile range

To our knowledge, scavenger receptor CD163 can be expressed on the surface of monocytes and macrophages, as well as in a soluble form in plasma. We thus determined the levels of sCD163 in the plasma of populations with different tuberculosis infection statuses. We observed significantly high levels of sCD163 both in ATB and LTBI groups, compared with those in the HC group (1329.0 ng/ml vs 844.3 ng/ml, *P* = 0.0004; 1304.0 ng/ml vs 844.3 ng/ml, *P* = 0.0216; Fig. 4a). However, there was no significant difference between the ATB and LTBI groups (1329.0 ng/ml vs 1304.0 ng/ml, *P* = 0.5045) (Fig. 4a). ROC analysis was also performed to evaluate the potential value of sCD163 in plasma for the differential diagnosis of PTB and HC. The area under ROC curve (AUC) was 0.7698 with an optimal cut-off value of 1260 ng/ml (sensitivity: 58.97%, specificity: 90.48%, Fig. 4b). Considering that TBP is the most common extra-pulmonary tuberculosis, we next examined the levels of sCD163 in the PE and plasma of patients with TBP. As expected, sCD163 levels in the lesion of TBP patients were almost twice those in plasma (2116.0 ng/ml vs 1236.0 ng/ml, *P* < 0.0001, Fig. 4c), and a similar result was seen in patients with MPE (1820.0 ng/ml vs 783.6 ng/ml, *P* < 0.0001, Fig. 4c). Notably, the levels of sCD163 in PE and plasma samples of patients with TBP were significantly higher than those in patients with MPE (2116.0 ng/ml vs 1820.0 ng/ml, *P* < 0.0001; 1236.0 ng/ml vs 783.6 ng/ml, *P* = 0.0040, Fig. 4c). Therefore, we further evaluated the value of sCD163 in PE and plasma for the differential diagnosis of patients with TBP and MPE. ROC analysis showed that sCD163 levels in PE and plasma for diagnosing TBP exhibited an AUC of 0.8418 and 0.8136, respectively, with a cut-off value of 1950.0 ng/ml (sensitivity: 64.29%, specificity: 100.00%, Fig. 4d) and 934.7 ng/ml (sensitivity: 77.27%, specificity: 80.00%, Fig. 4e), respectively.

### Relationship between soluble CD163 levels in plasma and disease severity in patients with pulmonary tuberculosis

**Fig. 5:**
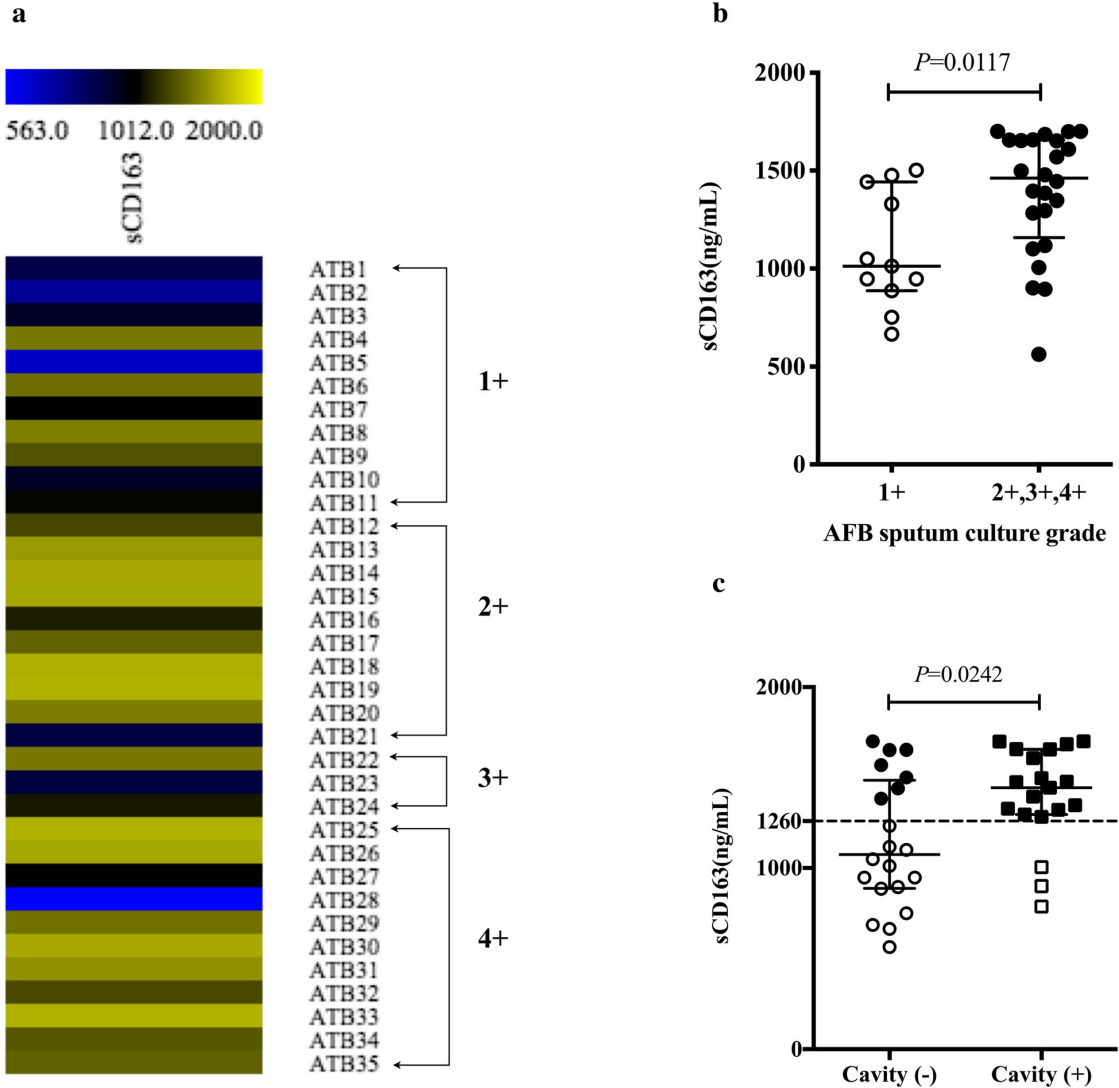
Relationship between sCD163 levels in plasma and disease severity. **a** Heat map of plasma sCD163 levels in patients with AFB sputum culture-positive PTB. **b** Plasma sCD163 levels were compared between 1+ and > 1+ groups. **c** Plasma sCD163 levels were compared between cavity (−) and cavity (+) groups, the cut-off value of 1260 ng/ml (dotted line) for the diagnosis of PTB was used as the threshold between sCD163 high (filled) and low (unfilled). Data are expressed as median with IQR and are analyzed using Mann-Whitney test. PTB, pulmonary tuberculosis; ATB, active tuberculosis; AFB, acid-fast bacilli; IQR, interquartile range

According to the grade of AFB sputum culture, PTB patients were divided into two groups: 1+ and > 1+ (2+, 3+, and 4+). Interestingly, we observed that the increased level of sCD163 in plasma was strongly associated with increased *M. tb* loads in sputum (1+ group: 1012.0 ng/ml, > 1+ group: 1461.0 ng/ml, *P* = 0.0117, Fig. 5a and b). In addition, among patients with PTB, approximately 48.7% (*n* = 19) showed cavity formation (Table 1). We further compared the sCD163 levels in plasma between cavity (+) and cavity (−) groups, and found an evident increase of sCD163 in cavity (+) group compared with that in cavity (−) group (1445.0 ng/ml vs 1075.0 ng/ml, *P* = 0.0242, Fig. 5c). In Fig. 5c, the cut-off value of 1260 ng/ml for the diagnosis of PTB was used as a threshold between sCD163 high (filled) and low (unfilled). Importantly, high sCD163 expression accounted for 16/19 (84.2%) in the cavity (+) group, but 7/20 (35.0%) in the cavity (−) group. These findings suggest a potential role of plasma sCD163 in predicting PTB disease severity.

### sCD163 levels in plasma decreased at different time points during anti-TB therapy

**Fig. 6:**
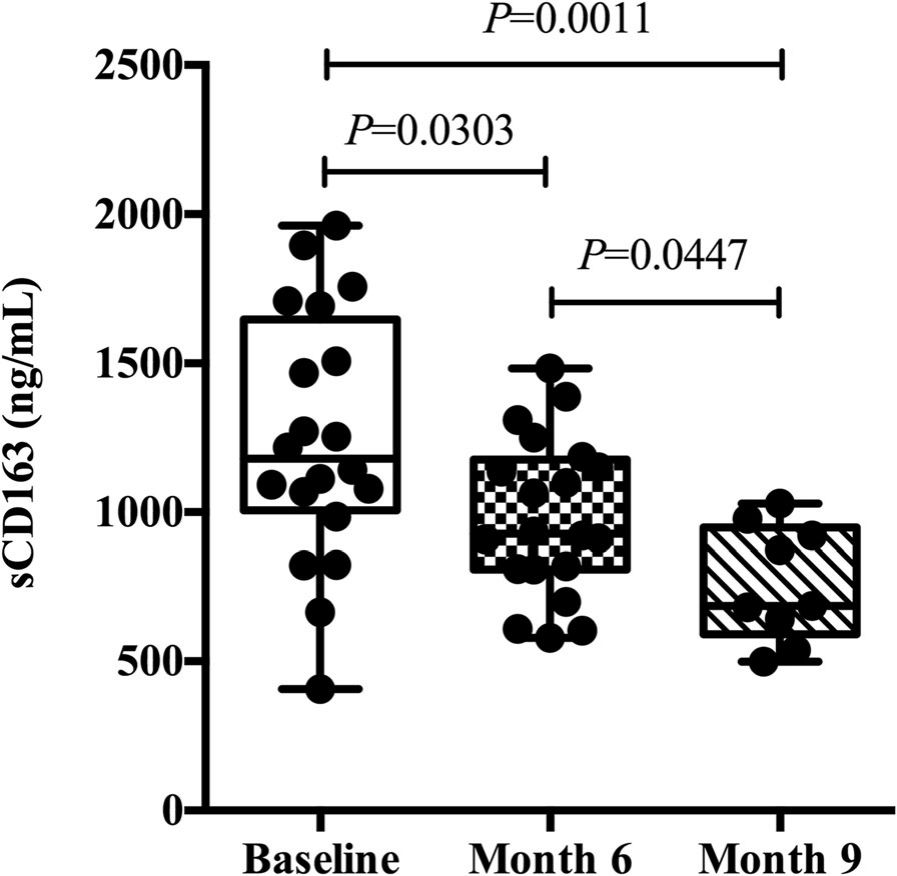
sCD163 levels in plasma were decreased in response to effective anti-TB treatment. Data are expressed as median and extremum, and are analyzed using Mann-Whitney test. TB, tuberculosis

We have previously found that increased plasma sCD163 levels were associated with disease severity (Fig. 5b and c). Next, we followed up with plasma samples of patients with TBP at month 6 and month 9 during effective anti-TB treatment. As expected, the results demonstrated that sCD163 levels in plasma were decreased significantly at various time points during anti-TB therapy, especially at month 9 of treatment, compared with that at the baseline (1180.0 ng/ml vs 685.9 ng/ml, *P* = 0.0011, Fig. 6). Furthermore, compared to the sCD163 levels during treatment for 6 months, they further decreased at month 9 of treatment (928.4 vs 685.9 ng/ml, *P* = 0.0447, Fig. 6). Therefore, changes in sCD163 levels might reflect changes in the burden of *M. tb*, which support the observations in Fig. 5.

## Discussion

In this study, we compared three major monocytes subpopulations between PTB patients and HCs, and found an increased frequency of CD14^+^CD16^+^ and CD14^−^CD16^+^ monocytes and a decreased percentage of CD14^+^CD16^−^ monocytes in patients with PTB. Currently, several studies have shown that changes in the profile of monocyte subsets during *M. tb* infection indicate bacterial persistence [14–16]. A previous study has similarly demonstrated that an increased proportion of CD14^+^CD16^+^ monocytes was associated with mortality in HIV-coinfected TB patients [17]. *M. tb* infection has been shown to induce expansion of peripheral blood CD16^+^ monocytes spontaneously undergoing late apoptosis [18]. However, in the context of TB, CD16^−^ (classical) monocytes as the dominant innate immune cells against TB contribute to the restriction of *M. tb* growth by rapid migration to infection sites and high production of reactive oxygen species (ROS) [16]. CD14^+^CD16^+^ monocytes exhibited higher phagocytic activity and lower antigen presentation compared to the CD14^−^CD16^+^ monocytes; additionally, this population was a major source of the immunosuppressive cytokine IL-10 [19]. Therefore, according to the characteristics of the above different monocyte subsets, our results indicated that an increase in non-classical (CD14^−^CD16^+^) and intermediate (CD14^+^CD16^+^) monocytes plus a decrease in classical (CD14^+^CD16^−^) monocytes might lead to the dissemination of *M. tb* infection and be involved in the immunological pathogenesis of TB.

CD163 is considered a specific marker of M2 macrophages [20]. We explored the expression of CD163 on different CD16^+^ monocytes subsets based on their CD14 expression. Surprisingly, scavenger receptor CD163 was mainly expressed on intermediate rather than non-classical monocytes, with a higher expression in PTB patients than that in HCs. Cougoule et al. also observed M2 markers including CD163 and CD206 augmented in the CD16^+^ subset compared to the CD16^−^ population, but they did not detect the expression of M2 markers in intermediate monocytes [12]. Indeed, they demonstrated that monocytes differentiated towards M2-like macrophages (CD16^+^CD163^+^MerTK^+^) depending on the IL-10/STAT3 signaling pathway in the context of TB, and that this phenotype rendered the host permissive to intracellular *M. tb* growth and an impaired ability to activate the Th1 immune response [12]. Furthermore, CD163^+^ monocytes can secrete both pro- and anti-inflammatory cytokines such as TNF-α and IL-4 during *Leishmania* and hepatitis C infection, which might interfere in instructing T cells and inhibit the killing of intracellular pathogens [21]. Thus, our findings indicated that high expression of CD163 on CD16^+^ monocytes in PTB patients might be involved in *M. tb* infection.

Additionally, sCD163 has been recently considered a novel soluble biomarker of monocyte/macrophage activation in pathological conditions [22–24]. Consistent with the membrane CD163 expression, our results show increased levels of sCD163 in ATB patients, which were particularly higher in PE than in plasma, suggesting that CD163^+^ monocytes might migrate to the site of infection in order to play an immunomodulatory role. This observation was consistent with the findings in patients with TB-associated immune reconstitution inflammatory syndrome (TB-IRIS), who exhibited high levels of sCD163 before anti-retroviral therapy (ART) and a worse prognosis [25]. Our findings revealed sCD163 as a potential biomarker in the diagnosis of ATB with a high specificity and low sensitivity, allowing the distinction of TBP from MPE. With an optimal cut-off value of 1950.0 ng/ml and 934.7 ng/ml, respectively, the sCD163 levels in PE and plasma showed AUCs of 0.8418 and 0.8136 in the diagnosis of TBP, respectively. Regrettably, sCD163 levels in plasma could not differentiate ATB from LTBI, whereas they could be used to distinguish ATB from HC with an AUC of 0.7698. Similarly, ROC analysis was conducted in TB patients and healthy subjects, showing a AUC of 0.78, but the differences between tuberculous and malignant PE were not analyzed in this study [12]. Indeed, many biomarkers such as adenosine deaminase (ADA), lactate dehydrogenase (LDH), and IGRA, have been investigated to distinguish TBP from MPE patients, with a large range variation in sensitivity and specificity [26–29]. Based on our results, the sCD163 levels in PE and plasma could be a useful biomarker for the diagnosis of TBP.

More importantly, we found a strong association between high sCD163 levels and TB disease severity in the present study. First, the increased levels of sCD163 in plasma were strongly linked to increased *M. tb* loads in sputum. Next, in the cavity (+) group, the proportion of high sCD163 (> 1260 ng/ml) expression (84.2%) was more than twice as large as that in the cavity (−) group (35.0%). Moreover, after effective anti-TB therapy, plasma sCD163 levels in patients with TBP were decreased significantly. A prospective cohort study followed up 113 verified TB patients, and demonstrated an association between high sCD163 levels (> 3950 ng/ml) and increased mortality [30]. Actually, the serum levels of sCD163 in PTB patients were restored to normal levels after 12 months of anti-TB treatment [12]. These findings suggest that sCD163 might be a predictive biomarker for TB prognosis.

## Conclusions

Taken together, our study indicates an increased frequency of CD14^+^CD16^+^ and CD14^−^CD16^+^ monocytes and a decreased CD14^+^CD16^−^ population in PTB patients. Both membrane and soluble CD163 are markers of monocyte/macrophage activation, which were increased in patients with ATB, especially in pleural effusions. Furthermore, sCD163 can be used to distinguish TBP from MPE patients with a high specificity. Importantly, there was a strong association between the high sCD163 levels and TB disease severity. We also followed up plasma samples of TBP patients and observed a significant decrease in plasma sCD163 after effective anti-TB treatment. Therefore, CD163 may offer a new insight in the diagnosis and prognosis of TB patients.

## Data Availability

The datasets generated and/or analyzed during the current study are not publicly available due to anonymity policy issues but are available from the corresponding author on reasonable request.

## Abbreviations

AFB: Acid-fast bacilli
ELISA: Enzyme linked immunosorbent assay
HC: Healthy control
IGRA: Interferon-γ release assay
LTBI: Latent tuberculosis infection
*M. tb*: *Mycobacterium tuberculosis*
MPE: Malignant pleural effusion
PBMCs: Peripheral blood mononuclear cells
PE: Pleural effusion
PTB: Pulmonary tuberculosis
TBP: Tuberculous pleurisy
